# Stabilizing family life after gastric bypass surgery

**DOI:** 10.1080/17482631.2017.1325674

**Published:** 2017-07-25

**Authors:** Ami Bylund, Eva Benzein, Anna Sandgren

**Affiliations:** ^a^ Department of Health and Caring Sciences, Linnaeus University, Kalmar and Växjö, Sweden; ^b^ Karolinska Institute, Department of Surgical Science at Danderyd Hospital and Department of Surgery, Ersta Hospital, Stockholm, Sweden; ^c^ Center for Collaborative Palliative Care, Department of Health and Caring Sciences, Linnaeus University, Kalmar and Växjö, Sweden

**Keywords:** Grounded theory, obesity, remodelling family patterns, weight loss, weight loss surgery

## Abstract

Weight-loss surgery requires lifelong lifestyle modifications for the maintenance of weight loss and health effects, and can affect both the individual and family. Earlier research indicates that the quality of social relationships has positive and negative influences on wellbeing and health. There is little research on family-life after a member has undergone gastric bypass (GBP) against obesity. Thus, this study aimed to develop a classic grounded theory about families with a member treated with GBP against obesity. The study design used classic grounded theory and included data from 16 interviews. Families’ shared a main concern of unexpected changes after GBP, resulting in the theory Stabilizing family life, explained as a social process to decrease uncertainty and find stability and well-being in family interactions. The social process develops differently which entail families: attaining unity, returning to old patterns, or disconnecting to find stability, depending on the discrepancy in expectations and knowledge. This is affected by the overall life situation, life-stage and relationship quality. The theory highlights unexpected change as a potential challenge for the family, as well as how they resolve this. Hence, the theory can be applied in care strategies for families. Identification of families needing support to stabilize family life after GBP requires further research.

## Introduction

Weight loss surgery is an established treatment against obesity to reduce comorbidity and mortality and requires lifelong lifestyle modifications for maintaining weight loss and accompanying health effects, and may affect both the individual and the family (Vidot, 2015). Earlier research indicates that social relationships influence health and wellbeing and may change post-surgery in both a positive and negative direction (Bylund, Benzein, & Persson, 2013; Meana & Ricciardi, 2008). Research regarding weight loss surgery has mainly focused on medical outcomes (e.g., weight loss and reduction in comorbidity, mortality) (Santry, Gillen, & Lauderdale, 2005) and less on psychosocial issues (Grimaldi & Van Etten, 2010). Moreover, there is a scarcity of studies from a family perspective in care provision to an adult family member undergoing GBP.

## Background

Obesity is defined as a chronic disease (AMA, 2013; WHO, 2010) and a lifelong struggle with a negative impact on health and quality of life (Aguilera, 2014; Schauer et al., 2012). So far, surgical treatment has been shown to be the only long-term solution for weight loss and reducing the number of comorbid conditions (Ayman et al., 2010). The most established and used method worldwide is GBP, which has proven to be effective, resulting in significant weight loss and increased quality of life (Livhits et al., 2011; SOReg, 2014). However, 10–25% of patients experience postoperative weight regain after about 2 years, for various reasons, ranging from lack of motivation and confidence to difficulties with managing food cravings (Engström & Forsberg, 2011 ; Peacock & Zizzi, 2011; Sarwer, von Sydow Green, Vettler, & Wadden, 2009). Previous studies have indicated that support from family members may result in improved weight loss maintenance (Aguilera, 2014; Stewart, Olbrisch, & Bean, 2010). Although weight loss surgery has shown good results, it is not a “quick fix”; it requires lifestyle modifications, so persistent weight loss and health effects can be fully appreciated (Vidot et al., 2015). Lifestyle modifications can affect both the individual and his/her family (Bylund et al., 2013; Willmer et al., 2015; Woodard, Encarnacion, Peraza, Hernadez-Boussard, & Morton, 2011) when they are faced with a new situation, as a result of GBP (Sogg & Gorman, 2008). However, research on how families handle lifestyle modification and associated physical and psychosocial changes is scarce (Grimaldi, 2010; Vidot et al., 2015).

Previous findings for patients with chronic illness and in recovery have shown that family members are often regarded by patients as the most significant persons in providing support (Al Mutair, Plummer, O’Brien, & Clerhan, 2013). From a caring perspective, a family member is a person regarded as family by the patient and acknowledges this status (Wright & Leahey, 2013). Using a systemic and holistic view of care means that both the patient and family are considered a unit; this means that if life circumstances alter for one family member, it also affects the other family members (Wright & Leahey, 2013). Family relationships can both enhance the health and wellbeing of all family members, or increase their suffering and negatively affect their health (Gruber & Haldeman, 2009; Wright & Leahey, 2013). In both circumstances, the family perspective might generate positive effects for both the patient and the family by becoming aware of their own resources in problem solving, and increases understanding for the situation (Vidot et al., 2015; Wright & Leahey, 2013). There is a limited body of knowledge of how family involvement can affect lifestyle modifications after GBP (Vidot et al., 2015), and how families come to terms with their life situation. By exploring families’ main concern and generating a theory to explain families’ behavioural patterns, tailored support can be developed according to families’ needs (Table I).

**Table I.** Overview of the theory “stabilizing family life”.

Waiting out	Figuring out	Remodelling family patterns	Refiguring	Responding to stabilizing family life		
				Attaining unity	Returning to old family patterns	Disconnecting
Silent observing	Attentive comparing	Negotiating	Attentive comparing	Acknowledging	Compromising change	Distancing
Holding on	Decoding	Prioritizing	Mapping	Reflecting	Holding on	Critiquing
	Mapping	Planning	Acknowledging	Expressing needs		Spending less time together
		Mimicking new behaviour		Engaging with each other		Making different choices

Therefore, this study sought to develop a classic grounded theory about families with a member who has undergone GBP, as a measure against obesity. The following research question guided this study: what is the main concern for families after GBP and how do they resolve it?

## Materials and methods

Classic grounded theory was used in this study. This method aims to conceptualize human behaviour (Glaser, 1998), specifically categorizing behaviour, and not people (Glaser, 1978). Grounded theory is a general method entailing the generation of concepts. In this study, these concepts are related to each other as a theoretical explanation for actions that resolve participants’ main concern (Glaser, 1998). Our study aimed to develop a substantive theory to explain the behavioural patterns of families with a member who has undergone GBP, as a measure against obesity.

### Participants and data collection

The study was conducted from September 2014 to June 2015. The setting was a university-affiliated bariatric centre in an urban area in central Sweden. The researcher asked two nurses specialized in obesity care, who were in charge of the follow-up visits after GBP, to recruit family members. The nurses were instructed to ask the patients at the 2-year follow-up visit about study participation and to invite at least one family member. Moreover, the nurses asked for permission for the researcher to call the patient and the invited family members for additional information and to schedule a time for the interview. For the purpose of this study, a family member is defined as a person identified by the patient as family. This includes biological relatives or those regarded as significant in patients’ lives. The inclusion criterion for patients was having undergone GBP surgery for obesity. Exclusion criteria were previous weight loss surgery and the inability to communicate in Swedish.

One interview was conducted with each of 12 patients, together with one to three additional family members, in all, 11 men and 17 women. Four of the 12 interviews were conducted individually because of difficulties with arranging for a suitable interview time with both a patient and a family member. Participants were aged 18–67 years; the length of their relationships ranged from 7 years to lifelong. Families consisted of partners, siblings, children, relatives and friends. The majority of the interviews were conducted at the families’ homes. Four interviews were, by the participants’ request, conducted in a secluded room at the clinic. Additionally, secondary analysis was conducted on data based on four family interviews (four men and four women) from a previous study, (Bylund et al., 2013). In line with the grounded theory concept of “all is data” (Glaser, 1978). In total, data from 36 participants were included (12 interviews and data from four previous family interviews).

The interviews began with an open question, with family members asked to talk about their present situation. This allowed family members’ experiences to unfold without being steered by preconceived questions. The interviews could be characterized as open conversations, as opposed to formal interviews. During the interviews, new questions and ideas emerged in relation to the study that were asked and explored in later interviews; for example, “Can you give me an example of an unexpected situation? How do you deal with that?” Later on, more specific questions were asked, to ensure saturation of the categories and concepts in the emerging theory (Glaser, 1998). This procedure forms part of theoretical sampling (Glaser, 1978). The interviews took 45–110 min and were mp3-recorded and subsequently transcribed. Field notes were taken, consisting of content and observational data (e.g., social interactions) from the interview.

### Data analysis

Data were collected and analysed simultaneously, using constant comparative analysis, with theoretical sampling guiding what data would be collected next (Glaser, 1998). After each interview, the transcribed interview and field notes were analysed and coded line-by-line. In this first open coding phase, *in vivo* codes (participants’ own words) were used to capture the incidents. Interviews were continuously compared with previous interviews and, during data collection, incidents were grouped into codes, and then into concepts. The analysis guided further data collection, which means that ideas that emerged during analysis became the focus for questions in subsequent interviews (Glaser, 1998). In the analysis, focus was on the behaviour of both patients and family members. Similarities, differences and variations within the data were detected. Open codes were then compared and questions asked by the researcher in relation to the data. “What is this study on?”; “What is happening in the data?”; “What do the incidents indicate?”; “What is the families’ main concern and how do they try to resolve it?” These questions enable the researcher to be theoretically sensitive and to conceptualize, as opposed to being stuck at a descriptive level (Glaser, 1998). The initial codes were then compared to generated concepts, to ensure that the data and the concepts were related. Through constant comparison, a core concept started to emerge. The core concept explains how participants resolve their main concern (Glaser, 1998). After identification of the core concept, the selective coding phase began. Further data were collected and coded using theoretical sampling, with a focus on concepts related to the core concept. To saturate data, secondary analyses from a previous family study were conducted, which led to further clarification of the concepts. Coding proceeded until theoretical saturation was achieved, meaning that no new concepts could be identified, and there was no longer variation within existing concepts (Glaser, 1998).

In the theoretical coding phase, the relationships between the concepts and the core concept emerged through sorting the memos to find the theoretical code(s) (Glaser, 1998). Memos were written throughout the analysis, to capture ideas and clarify hypotheses relating to the concepts and their interconnection with each other (Glaser, 1998). By writing memos on memos, one can increase the level of abstraction from data to codes through constant comparison, to reach an abstract conceptual level and discover behaviour patterns. The types of theoretical codes generated may vary (e.g., processes, causes, contexts and conditions) (Glaser, 1978, Glaser, 2010). The theoretical code in this study emerged as a social process with different strategies. After formulation of the substantive theory, a literature review was conducted, as recommended for Classic Grounded theory (Glaser, 1998). Since all constitute data (Glaser, 1998), the literature was used as data in the constant comparative process. The literature review enriched the concept meaning and the generated theory.

#### Ethical considerations

Permission to conduct the study was obtained from the head of the department and the hospital research committee. Ethical approval was obtained from the Research Ethics Committee at Linköping University, Sweden (Approval number 2011/157–31), in accordance with the Declaration of Helsinki (WMA, 2015) and Swedish law (SFS 2008: 192). After receiving information about the study in writing and verbally, and about confidentiality, participants signed an informed consent form.

## Findings

Facing unexpected changes in the family life emerged as the main concern for the families. Even though families are aware of the physical consequences of GBP, they are surprised and challenged by how the unexpected changes affect the family. “If we knew the consequences of the change, we would not have taken the decision regarding GBP so lightly.” Families’ preconceived expectations are that intrapersonal changes and a new lifestyle regime are individual issues; however, the whole family undergoes unexpected change. Examples of unexpected change are an altered physical appearance and possible GBP side effects (dumping syndrome, characterized by, for example, abdominal cramping, nausea, a rapid heartbeat, dizziness, or other problems such as diarrhoea, difficulty eating because of abdominal pain and depression). Changes that also affect the whole family could be psychosocial and may include mood swings (e.g., a short temper, frustration, sadness and tearfulness), being more assertive and energetic, and perceiving a higher degree of acceptance from others. These changes affect health, wellbeing, and interactions (e.g., communication patterns, habits and routines within the family). Family members’ response to changing attitudes by the extended family and friends could be a challenge. Family members may be surprised and confused by their own reaction of resentment and frustration when people who previously ignored their family members when they were obese now give them a lot of attention and affirmation. Increased attention from extended family and friends also increases disruptions in the interaction between family members (e.g., jealousy, insecurities and a shift in dynamics). Different expectations and knowledge regarding change after GBP within the family increase uncertainty and unpredictability, regardless of whether the change is perceived as positive or negative.

The theory, *stabilizing family life*, explains a process of decreasing uncertainty and unpredictability when facing these unexpected changes, in order to find stability and wellbeing in interaction and routines. The theory consists of the following stages: initially, the *waiting out stage*, which entails expecting unexpected change to subside; followed by *figuring out unexpected changes*, to recognize and predict situations; *remodelling family patterns*, to find new ways to cooperate; and lastly, *modifying family life*, in which attaining unity, returning to old family patterns, or disconnecting is used. During the whole process, *protective shielding* is a commonly used strategy

Family life can be stabilized in different ways, depending on the discrepancy between family members, in terms of knowledge and expectations, the degree of flexibility towards change, and the quality of dynamics such as communication, problem-solving skills and ways of relating to each other. The degree of discrepancy influences how families perceive the degree of challenge, and therefore triggers different resolving loops in the theory, namely, the *integrating loop*, which results in *attaining unity* for the family, or *disintegrating loop*, which result in *returning to old family patterns or disconnecting*. These resolving loops explain why and the manner in which the families are using different stages and strategies to stabilize family life and the GBP outcomes. The families can move back and forth between the stages in the process, depending on whether something new happens that challenges the stability of family life, such as complications, new unhealthy eating or drinking habits or another illness.

### The theory

#### Waiting out

In order to handle the changing dynamic, families first go through a period of *waiting out*, which entails waiting for the effects of the unexpected change to subside. This is done through *silent observing* while *holding on* to established routines and habits to preserve stability. Silent observing means that one is observing interaction and ways of handling daily routines (e.g., meals and socializing with friends), but regards this as a temporary situation. This is based on being involved in previous experiences of trying to make lifestyle modifications (e.g., through behavioural treatment for obesity). However, tensions often increase in the dynamic, leading to disturbances in the interaction in relation to everyday routines, diet and lifestyle changes, which leads to nagging or frustration. These challenges may work as triggers towards finding a resolution through *figuring out* unexpected change.

#### Figuring out

*Figuring out* unexpected changes is a way of learning how to recognize and predict situations and intrapersonal changes within the patient. These changes could result in tension and disturb the social interaction. Figuring out is done through *attentive comparing, decoding* and *mapping*.

*Attentive comparing* means linking unexpected change to previous experiences. This is done by comparing current developments with previous, well-known situations, to identify differences (e.g., in social situations or at mealtimes). Attentive comparing also helps in *decoding* unexpected change, trying to understand when and why unexpected change happens. Attentive comparing is done through observing and experiencing unexpected change. Through experiencing what does not work anymore, decoding new situations become a learning strategy for the families. For example, “we now have to consider that if we do not eat regularly, our family member feels unwell”; or in interpersonal interaction situations, being assertive leads to new tensions in the family. *Mapping* begins after decoding and is a way of becoming aware of the possibilities and limitations brought about by change; for example, how to avoid triggering events that could otherwise cause concern in the family or discovering new possibilities in shared parental responsibilities. These could entail, for example, family members being informed that the patient can now be actively involved in everyday activities in a new way and on the same terms as other family members. Figuring out unexpected change is a prerequisite for effective manoeuvering of *unexpected changes* and moving on to the next stage, *remodelling family patterns.*

#### Remodeling family patterns

As unexpected changes progressively become easier to predict in life situations, families have the energy to remodel family patterns to achieve stability and wellbeing. Remodelling occurs over a long period of time and enables families to find new ways of cooperating. Remodelling occurs through *negotiating, prioritizing, planning and mimicking new behaviour*.

Because of increased tension and changing family roles, triggered by unexpected change in family situations, there is a need for negotiating family routines and rules. *Negotiating* is used to seek agreement about altered needs, to become more active and involved as, for example, a parent, partner or a friend. After GBP, the treated family member gains energy and is eager to get more involved in sharing responsibilities in family life, as well as spending more time outside the family, to develop a social network. This leads to a need to negotiate new agreements regarding roles, routines and interactions. Negotiating makes it easier to deal with challenging situations, such as the development of new symptoms or interaction patterns. Negotiating occurs either verbally or in writing, entailing indications on how to solve family situations perceived as challenging.

Reaching agreements leads to new *prioritizing*, which is done through mutual reflection on choices that support interaction and wellbeing. These include attending to family needs first, by focusing on achieving wellness through eating together, increasing awareness regarding healthy eating habits by children, and paying attention to each other’s habits, that might have a negative health impact. With prioritizing follows a need for *planning*, to enable the breaking down of activities to foreseen, manageable challenges. For instance, making detailed preparations for family activities, to make a situation easier to handle, and maintaining healthy choices (e.g., dividing parental tasks, making time for regular meals, physical activities, preparing food/snacks to avoid resorting to last-minute solutions of fast food).

*Mimicking new behaviour*, which works as a way of accommodating changes, occurs as a result of *planning*. Families use mimicking to make lifestyle modification a natural part of everyday life. Sharing the same lifestyle or experiences of GBP is considered a solution for better understanding of each other. For instance, in families where obesity is a shared health concern, other members consider undergoing GBP as a way of living and sharing the same lifestyle terms, since it has improved another member’s health. *Mimicking* equalizes relationships within families, aiding cooperation regarding habits, such as the adoption of similar eating habits and family members trying to alter their lifestyles together. Mimicking leads to the refiguring of families’ changed conditions, specifically with regard to altered family positions, dynamics and problem solving.

#### Refiguring

*Refiguring* is done to identify which strategy to use to handle unexpected change. This stage is similar to that of *figuring out*, but proceeds faster because families have experienced different situations with unexpected changes, and are therefore prepared and have gained the knowledge to handle these situations. In this stage, unlike in *figuring out*, there is a mutual awareness of the character of the change (e.g., mood swings, the establishment of new roles and new family communication patterns). *Refiguring* is done through *attentive comparing, mapping* and *acknowledging. Acknowledging* means accepting unexpected changes as part of family life. Accepting entails a shift in focus to perceiving unexpected change as a family concern instead of an individual issue.

#### Stabilizing family life

The above presented a process entailing four stages, which takes three different directions: *attaining* unity; *returning to old family patterns; or disconnecting* to stabilize family life, depending on how families perceive the challenge of unexpected change and the ability to use the different strategies.

*Attaining unity* means getting all family members involved in co-creating stability and wellbeing together. This is done by *acknowledging change and reflecting* on possibilities (e.g., skills and knowledge) and limitations (e.g., perceived symptoms after GBP and risky situations), and *expressing needs*, *engaging in each other*. Acknowledging change is done by accepting new needs as part of family life and co-creating a new mutual life of setting goals together; examples thereof are having children, moving to another part of the country, or starting to work again after years of unemployment. Part of *acknowledging* is *reflecting* together on the limitations (e.g., dumping syndrome, trouble eating, increased alcohol intake and tense family situations) and possibilities, thus contributing towards the generation of different solutions for everyday challenges. By reflecting together, families start *expressing* physical and/or emotional needs through more straightforward communication, such as asking for time to be with friends or requesting more equal distribution of childcare or household work.

Another way is *engaging* with each other in a new flexible way, by *working more as a team*. Teamwork increases stability and wellbeing through actively sharing day–to-day responsibilities, setting up mutual everyday goals together, the family engaging in leisure activities (increased interaction) and leading active lives (increased physical activity) together, and playing with one’s children. Families can work around challenges by acknowledging change and expressing their needs; for example, feeling that the partner spends too much time outside the home or getting too much attention. Being older and having a longer established relationship (over 7 years) with higher degree of understanding for each other’s needs seemingly facilitates an open communication environment. Attaining unity is a desirable stage, since families come to a new realization and find a shared resolution to a way of living that stabilizes family life after unexpected change.

*Returning to old family patterns* implies an inability to stabilize family life and accommodate the changes together. It is a return to the status quo and constitutes no contribution towards the family’s wellbeing and interaction patterns in the new situation. Returning to old family patterns is done by *compromising changes*, which refers to trying to keep changes at an individual level and avoiding influencing the family, such as surrendering the role of the main caregiver to the person who used to be in charge of monitoring health and wellbeing. *Holding on t*o well-known routines and dynamics is another way of establishing stability and a sense of control. This occurs when change is too challenging; for example, lifestyle modifications or psychological changes such as mood swings. It can also occur when the conditions for and consequences of change are questioned. The return to old patterns may be due to additional challenges such as illness, financial strain, rigid relationship patterns and less engagement in joint recreational eating, which was previously a way of showing appreciation to one another and spending quality time together.

*Disconnecting* refers to detachment from relationships, as a solution, which is done by *distancing* oneself or *resigning*. This could occur when the changes are too different from pre-GBP family life.

Distancing involves decreased communication, such as not talking about family issues or partaking in shared social gatherings and activities together, and is done through *critiquing* and *spending less* time together. *Critiquing* means that family communication may change and entail critical comments about weight loss, psychological changes and changes in interpersonal relationships. Sharing less time means spending less time together as a family and brings about tense and stressful situations, such as increased disagreement on how to solve problems related to everyday tasks, decreased emotional support and less support regarding the maintenance of lifestyle modifications. Seeking support from friends outside the family in making individual decisions about family life increases distancing. Distancing can lead to either *refiguring out unexpected change*, which means that the family tries to identify what happened or turns to *resigning. Resigning* means giving up trying to seek stability together (e.g., separation). It becomes a solution when interrelations are characterized by family members going in different directions to seek stability and wellbeing, with one side trying to maintain the status quo and the other striving towards changes in roles, behaviour and interactions. Resigning may instigate the return to old family patterns or the choice to live separate lives. Factors influencing disconnection in a bid to find stability and wellbeing include different opinions regarding the extent to which change should affect the family. Moreover, having preschool or school-going children seemingly increases the likelihood of perceiving unexpected change as a challenge, as opinions may differ regarding lifestyle modifications for the children, too. Other influential factors may be finances, previous relationship problems, health issues and the surrounding family members’ response to change.

#### Protective shielding

Protective shielding is an ongoing strategy which is used in different ways throughout the process of *stabilizing family life*. It is done to attain control and protect oneself and/or the family from situations that may cause suffering and disturbance (e.g., destructive eating habits or increased alcohol use). The intensity of protective shielding depends on the extent to which unexpected change triggers disruption. Protective shielding is done by *preventing consequences of change* and *opposing* change.

*Preventing consequences of change* is used to decrease perceived challenging situations, such as monitoring behaviour or ensuring that lifestyle modifications are adhered to (e.g., eating correctly and timeously). In interactions, *preventing consequences of change* occurs through holding back, not speaking one’s mind about perceived unacceptable habits and emotions. These could entail keeping quiet, despite concerns about unhealthy habits. Such concerns might include lack of self-care, demonstrated by not eating, eating too much, and due to feeling offended by negative remarks, withdrawing from social interaction, to avoid causing tensions in the relationship. Withdrawing may, in the end, result in *disconnecting* from each other because of the failure to find a joint solution.

*Opposing* provokes a reaction seeking to bring a challenging situation into the open. This appears when interrelations become tense after both a person changing psychologically (e.g., being short-tempered, energetic and outgoing) and a change occurring in interpersonal relationships. Opposing may serve as a way of holding on to old ways, to regain a sense of belonging together. It can work as a strategy to constrain behaviour perceived as unhealthy or to challenge interrelations.

#### Resolving loops

The theory, *stabilizing family life*, focuses on how families try to attain stability and wellbeing through different stages and can be explained through two resolving loops, namely, integrating and disintegrating loops. Resolving loop work as an explanation model on how the use of different strategies in the process leads to different directions in an attempt to resolve unexpected change. In other words, resolving loops can be seen as families responding differently to stabilizing family life.

An *integrating loop* means that families are dynamically in harmony with each other through communication and flexibility, striving in the same direction, which facilitates the stages of *figuring out and remodelling family patterns*. Unity occurs when families can adapt well to change. *Attaining unity* can be maintained through ongoing remodelling, by incorporating additional changes in the family interaction. In an integrating loop, *protective shielding* prevents situations of vulnerability, which otherwise create disruption and negatively affect wellbeing. Protective shielding seems to be a part of family life when lifestyle modifications have become a way of life. *Opposing* is used as a way of initiating the resolution of challenging situations and leads to *remodelling family patterns* and later on leads to *attaining unity*. The integrating loop can turn into a disintegrating loop if continuous remodelling does not occur.

A *disintegrating loop* entails that families come across unexpected changes from different starting points, and may be resolved in two different ways, namely, *returning to old family patterns* or *disconnecting*.

*Returning to old family patterns* is a resolution when only part of the family wants to undergo changes, *resigning* then works as a way to keep the family together to diminish disagreement regarding changes. This is mainly achieved by going back to well-known routines, roles and positions to minimize changes that might bring uncertainty in family dynamics. *Disconnecting* as resolution is used when families discontinue *remodelling* together, for example, when family members pull in different directions regarding everyday life, including problem solving, lifestyle choices and future aspirations. Failure at remodelling (due to the lack of *negotiating*, *prioritizing and planning*) leads to a reduced sense of mutual understanding and an increase in feelings of being left out. Families are divided into subgroups, making stability and wellbeing harder to achieve for the whole family. Failure to achieve remodelling increases stress and is resolved through last-minute solutions with limiting consequences (e.g., interruption of ongoing activities and cancelling or avoiding activities). Alternatively, remodelling attempts can be too strenuous because of different starting points and changes occurring in different areas (interactional or lifestyle changes), especially given additional challenges (e.g., financial difficulties or another illness). Disintegrative loops seem to develop when family members have diverging expectations, knowledge and perceptions of strained family dynamics, which may be accompanied by and increase the inability to integrate changes into family life. A disintegrative loop can either start to develop early in the process, during figuring out, or later on in the remodelling phase leading to a situation wherein family members do not work and communicate around change, which leads to challenges and additional stress and tension in families’ interaction and everyday life situations.

## Discussion

The key finding in this study was the families’ main concern of facing unexpected changes after GBP. The developed theory was the process of stabilizing family life that explains how families try to resolve unexpected change. This process develops into either an integrative or a disintegrative loop depending on different expectations, knowledge and different perceptions of strained family dynamics within the family. The theory also illuminates the need to include a family perspective when preparing for lifestyle changes related to GBP.

### An individual decision with a family impact

The finding of families’ main concern of unexpected change is in accordance with previous research with patients who indicated experiences of major changes in daily life after GBP, accompanied by decreased stigma in social situations, but also unexpected difficulties and feelings that complicated close relationships (Lier, Aastrom, & Rørtveit, 2016). The core concept, *stabilizing family life*, can be viewed from an individual level, based on previous research that showed that persons who have undergone GBP may have to handle novel interpersonal situations (Sogg & Gorman, 2008) that can contribute towards psychological distress (Gilmartin, 2013). The decision of GBP was taken by the individual alone, with the expectation that this was a change that would not have an impact on the family. Approaching from a systemic perspective, change in one member has an effect on other family members (Wright & Leahey, 2013). The level of discrepancy between family members’ expectations and life situations, on the one hand, and the unexpected change, on the other, had an impact on how the process developed into different resolution patterns, namely, attaining unity, returning to old family patterns and disconnecting. Moreover, the overall family situation and health played a role (e.g., new symptoms) that may disturb the ability to perform daily activities, or may trigger a disturbance in relationships, hampering cooperation within the family.

### Families ability to communicate and changing together

The theory, *stabilizing family life*, implies that *facing unexpected changes* is also a family matter. Our theory explains how families try to stabilize their new situation by *figuring out unexpected change* make predictions and engage in *remodelling family patterns* to a situation with new challenges, depending on their (families’) ability to communicate about unexpected change and co-create strategies. According to the family system theory, in order to adjust to change, family members need to change together so they can discover new dynamics and attain wellbeing (Wright & Leahey, 2013). Earlier research has shown that family and peer support is important in the establishment or attainment of lifestyle modifications and wellbeing, as well as the maintenance thereof (Ogle Park, Damhorst, & Bradley, 2016; Stewart et al., 2010; Wykowski & Krouse, 2013).

*Remodelling family patterns* was affected by families’ degree of understanding of change and degree of communication. Remodelling together made it easier to strengthen the interrelationships within the family, also facilitating *attaining unity* and the maintenance of lifestyle modifications. According to research, individuals often experience a positive change in their relationships after undergoing weight loss surgery (Clark et al., 2014; Sarwer, Wadden, & Fabricatore, 2005). Wedin et al. (2014) have reported that being married was 7.1 odd ratio with sustained weight loss. However, when the family relationship is threatened by change, family members try to resist this, with negative influences on both health outcomes and interrelationships (e.g., weight regain and relationship distress (Andrews, 1997, Ferriby et al., 2015). Wedin et al. (2014) identified a need to examine the quality of the relationship and how that affects outcomes e.g., weight loss and quality of life after weight loss surgery. Ferriby et al. (2015) found evidence for that some families’ relationship quality deteriorate after surgery. The present theory indicates that the quality of relationship affects stability and wellbeing on a family level. *Remodelling family patterns* is more difficult when there are substantial discrepancies within the family, with regard expectations and knowledge regarding the demands and implications that *unexpected change has on* interrelations and the management of day-to-day life. This is in accordance with earlier research on the individual level, which showed that unrealistic expectations and lack of support may negatively affect self-care management in the form of diet and physical activity, as well as the maintenance thereof (Moore & Cooper, 2015; Sharman et al., 2015). Families’ involvement and negative expression of support can be obstructive, depending on the relationship quality and conflict resolution abilities (Mayberry & Osborn, 2014; Rosland, Heisler, & Piette, 2012).

### Integrative or disintegrative loop in relation to first and second order change

In the presented theory, family resolution was explained as developing in a disintegrating or integrating loop characterized by either the return to old patterns, as only one family member changed, or joint development of new patterns. This is in line with Watzlawick, Weakland, and Fisch’s (1974) concepts of first- and second-order change. First-order change means that the system’s structure and process remains the same, even though roles and circumstances change—the character of the relationships and the structure remains intact. Second-order changes roles, relationships and the system’s structure—rules, boundaries and possibilities (Watzlawick et al., 1974). Change requires giving up familiar routines and can affect family members’ roles and standing (Watzlawick et al., 1974). Thus, our theory can be seen as entailing different strategies meant to overcome barriers of change and to develop a new structure of interaction. The concepts, the disintegrating or integrating loop, can be viewed as an expression of first- or second-order change in the family system. Depending on how the process of *stabilizing family life* develops in an integrating or disintegrating loop, there may be a need to identify families that would benefit from a family intervention, so as to enhance awareness about the resources and limitations within the family.

### Family perspective as a complement to an individual focus

Clinical care has mainly focused on the individual patient, giving less attention to socio-ecological factors (e.g., family resources, support and family functioning) (Rolland, 2015) within which patients introduce lifestyle modifications such as diet and physical activity. Even though earlier findings report that patients are satisfied with weight loss and quality of life after weight loss surgery, a substantial number of patients fail to achieve weight loss, regain weight or experience challenges in close relationships (Christou, Look, & Maclean, 2006; Groven, Råheim, & Engelsrud, 2013; Lier et al., 2016; ; SOReg, 2014). There is a need for further research on the identification of family factors that are important in maintaining lifestyle changes and the stability and wellbeing of the family after weight loss surgery.

#### Methodological considerations

Classic grounded theory (CGT) was chosen as an appropriate method, as it is often used in unexplored areas (Glaser & Strauss, 2006[1967]). Using CGT without a predefined question facilitated the discovery of the main concern, which could have been more difficult with use of methods with predefined problems. The theory of stabilizing family life is not conclusive and reflects one pattern of behaviour. There may be a variety of behaviours to use and needs to be explored. A possible limitation of the study was the role of the nurses who had initial contact with prospective participants, who could therefore be seen as gatekeepers, with the potential to avoid including some prospective participants in the study. However, there was no alternative way of gaining access to participants. Another possible limitation is that families that were not satisfied with their relationships may have declined participation in the study. This would suggest a bias towards families with satisfying relationships. However, some of the participating families stated they were considering separation.

In CGT, fit, relevance, workability and modifiability are quality-judging criteria for a theory (Glaser, 1978, 1998; Glaser & Strauss, 2006[1967]). Fit refers to the extent to which theory fits the studied situation and if categories explain patterns of behaviour (Glaser & Strauss, 2006[1967]). To achieve theoretical fit, conceptual memos were written and sorted in the analysis, to help identify the relationship between incidents and concepts, and between concepts and core concepts. Relevance addresses whether the theory is relevant to the studied area. To address relevance, which in this instance was the main concern of the participants, data were collected and coded until no new information emerged. Workability refers to whether the theory is identifying and explaining how the main concern is resolved (Glaser & Strauss, 2006[1967]). The analysis focused on finding a core concept that comprehensively explained how the main concern was resolved. Modifiability refers to whether the theory is flexible and modifiable when new data are added (Glaser, 1978, 1998). The presented theory is developed from data collected in the context of families who stay with a member who has undergone GBP against obesity. However, since grounded theory should be abstract in relation to time, place and people, the theory, *stabilizing family life*, might be of relevance after modifications in other substantive areas (e.g., management of chronic illness) and contribute to an understanding of the process of stabilizing family life. Further research is needed to explore if the theory fits in other contexts and instances wherein new concepts could emerge, for modification and enhancement of the theory’s fit.

#### Conclusions and implications

This theory may contribute to increased knowledge regarding how families stabilize family life and accommodate unexpected changes after GBP. This theory highlights the influence of family factors (i.e., interaction, problem solving and caring) on stability, wellbeing and lifestyle modifications. Failure to focus on how the whole family deals with changes after GBP may make it difficult to explain and, therefore, improve aspects of the quality of interactions and wellbeing, including health outcomes (e.g., lifestyle modifications). Family-based interventions may be of value to families that struggle to deal with and adapt to unexpected changes and lifestyle modifications. For other families, being involved and informed from the beginning might be sufficient to stabilize the new life situation. Further research must explore whether more awareness of pre-existing knowledge and expectations influences the reaction to unexpected changes. It is also necessary to explore ways of identifying families that could gain from more knowledge prior to weight loss surgery as treatment for obesity.
